# Predictive value of D-dimer in assessing the risk of pulmonary embolism (PE) in Covid-19

**DOI:** 10.5339/qmj.2024.qitc.6

**Published:** 2024-03-24

**Authors:** Muhammad Yousaf, Ahmad A. Abujaber, Salah Almughalles, Merlin Marry Thomas, Mansoor Ali Hameed

**Affiliations:** 1 Hazm Mebaireek Hospital, Hamad Medical Corporation, Doha, Qatar; 2 Weill Cornell Medicine-Qatar, Cornell University, Doha, Qatar Email: myousaf3@hamad.qa; 3 Hamad General Hospital, Hamad Medical Corporation, Doha, Qatar

**Keywords:** Covid-19, D-Dimer, Pulmonary Embolism, Venous thromboembolism

## Background

Venous thromboembolism (VTE) is a well-known complication associated with Covid-19. Multiple studies have explored the correlation between various biomarkers and VTE in individuals with Covid-19. We published a study on pulmonary embolism (PE) in Covid-19 (n = 193),^1^ which reported an incidence of PE in 21.8% (n = 42) cases. Only an elevated D-dimer was found to be associated with PE. We conducted a post hoc analysis to assess the predictive ability of D-dimer for PE.

## Methods and Results

**Figure 1.**
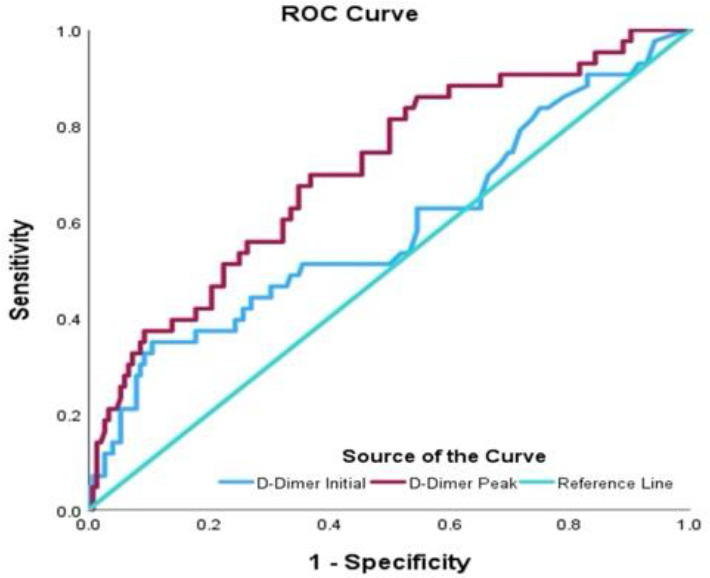
The area under the curve for peak D-dimer levels demonstrated acceptable discriminatory power.

The mean age of our sample was 51.48 ± 12.8 years and 67% had critical Covid-19 pneumonia. Binary logistic regression was used to assess the predictive ability of D-dimer at two distinct time points: at baseline (upon admission) and at peak during hospitalization. The logistic regression analysis revealed a notable association between peak D-dimer levels and the likelihood of PE occurrence, but no association between baseline D-dimer levels and PE occurrence. The area under the curve for peak D-dimer levels demonstrated an acceptable discriminatory power of 0.71 (Figure 1). Sensitivity, specificity, and positive and negative predictive values for peak D-dimer levels for predicting PE were 38%, 86%, 64%, and 64% respectively. The chi-square test identified a D-dimer cut-off point of 13.22mg/LFEU (normal D-dimer range 0–0.46 mg/LFEU). The chi-square test indicated a significant association (Table 1): 63% of patients with PE had a peak D-dimer level > 13.22, compared to 36.4% with a D-dimer level ≤ 13.22 (p-value < 0.05). D-dimer levels > 13.22 showed a likelihood ratio for developing PE of 1.782 (CI 1.652–1.922), providing robust support for a significant association between elevated D-dimer levels and the occurrence of PE in Covid-19.

## Conclusion

Peak D-dimer values may play a role in predicting PE in Covid-19. There are other similar studies with variable results.^2^ The potential of D-dimer prediction models to determine the urgency of diagnostic imaging and aggressive thromboprophylaxis regimens needs to be evaluated in prospective studies.

## Conflict of Interest

The authors have no conflicts of interest to declare.

## Figures and Tables

**Table 1.** Relationship between peak D-dimer values and PE occurrence.

			**CTPA Outcome**		
			PE	Non-PE	Total
**D-Dimer Peak**	**D-Dimer Peak > 13.22**	Count	28	55	83
		% with CTPA outcome	63.60%	35.70%	41.90%
	**D-Dimer Peak ≤ 13.22**	Count	16	99	115
		% with CTPA outcome	36.40%	64.30%	58.10%
**Total**		Count	44	154	198
		% with CTPA outcome	100.00%	100.00%	100.00%
